# RC-Effects on the Oxide of SOI MOSFET under Off-State TDDB Degradation: RF Characterization and Modeling

**DOI:** 10.3390/mi15020252

**Published:** 2024-02-07

**Authors:** Alan Otero-Carrascal, Dora Chaparro-Ortiz, Purushothaman Srinivasan, Oscar Huerta, Edmundo Gutiérrez-Domínguez, Reydezel Torres-Torres

**Affiliations:** 1 Instituto Nacional de Astrofísica, Óptica y Electrónica (INAOE), Puebla 72840, Mexico; alanyacceb@gmail.com (A.O.-C.);; 2 GlobalFoundries Inc., Malta, NY 12020, USA

**Keywords:** SOI, MOSFET, reliability, gate leakage

## Abstract

Based on *S*-parameter measurements, the effect of dynamic trapping and de-trapping of charge in the gate oxide, the increase of dielectric loss due to polarization, and the impact of leakage current on the small-signal input impedance at RF is analyzed and represented. This is achieved by systematically extracting the corresponding model parameters from single device measurements at different frequency ranges, and then the methodology is applied to analyze the evolution of these parameters when the device is submitted to non-conducting electrical stress. This approach not only allows to inspect the impact of effects not occurring under DC conditions, such as the current due to the time varying dielectric polarization, but also to clearly distinguish effects in accordance with the functional form of their contribution to the device’s impedance. In fact, it is shown that minor changes in the model of the gate capacitance by including additional resistive and capacitive components allows for an excellent model-experiment correlation up to 30 GHz. Moreover, the accuracy of the correlation is shown to be maintained when applying the proposal to the device under different gate-to-source bias conditions and at several stages during off-state degradation.

## 1. Introduction

Silicon-on-insulator (SOI) is a mature technology for radiofrequency (RF) applications due to its robustness against undesired device coupling and reduced parasitics when compared to the bulk metal-oxide-semiconductor (MOS) approach [1,2,3,4]. Furthermore, partially depleted SOI (PD-SOI) MOS field-effect transistors (MOSFETs) outperform their bulk counterparts without requiring the strict tolerances in substrate thickness associated with the more advanced fully depleted devices [5]. This has even allowed the implementation of microwave power amplifiers (PAs) using PD-SOI technology [6,7], where reliability issues become very relevant [8]. In this regard, the effects introduced by the generation of interface traps in the thin oxide are of particular interest due to the increased gate leakage current (*I*_G_) that may lead to the critical damage of transistors [9]. For this reason, time-dependent dielectric breakdown (TDDB) tests play a key role during device development and optimization [10].

Classical TDDB experiments are based on direct-current (DC) measurements that allow for monitoring the increase of *I*_G_ as electrical stress is applied to the device’s terminals [11,12,13,14]. Actually, there exist two main transistor states during which stress is applied: (i) while biased in the inversion region, and (ii) in channel depletion conditions. The first one is known as an on-state condition, whereas the second is referred to as either an off-state or non-conducting (nc) condition [15,16]. In general terms, degradation mechanisms occurring under these two conditions are relevant in current applications, but analyzing those becoming apparent for the nc state is of particular importance when studying the reliability aspects of PAs [17,18]. This serves as a motivation to carry out TDDB analyses considering off-state operation.

Hence, the change in the characteristics of transistors should be carefully quantified to assess the impact of off-state degradation on performance. For this purpose, several authors have proposed using RF measurement techniques to analyze the degradation in the response of the devices when carrying out TDDB tests [19,20]. In this regard, some RF measurement-based studies have been dedicated to identifying the effect of electrical stress on gain and cutoff frequency [21], on mobility and extrinsic parasitics [22], and, up until recently, also on the device’s input impedance [23,24]. In fact, since, under typical operation, the input impedance is that associated with the gate-to-ground path, interesting information about the change in the oxide characteristics can be acquired when analyzing this parameter [25].

Based on this discussion, this paper presents an RF modeling and parameter extraction methodology that allows for representing the input impedance of PD-SOI MOSFETs. Therefore, the resistive and capacitive parasitics that become accentuated within the gate oxide with degradation time are determined, which in turn allows to obtain the effective interface capacitance and trap time constant (*C*_trap_ and *τ*_trap_, respectively). This enables the indirect quantification of border traps generated during a TDDB degradation test. Furthermore, the enhanced model presented here can be used to inspect, through circuit simulations, the impact of the thin oxide degradation on the performance of analog and digital circuits using well-known methodologies such as those presented in [26,27]. This aids in predicting the conditions that might yield critical damage on actual circuits used at radiofrequencies [19].

## 2. Description of Devices and Experiments

### 2.1. Fabricated Devices and Test Fixture

**Figure 1:**
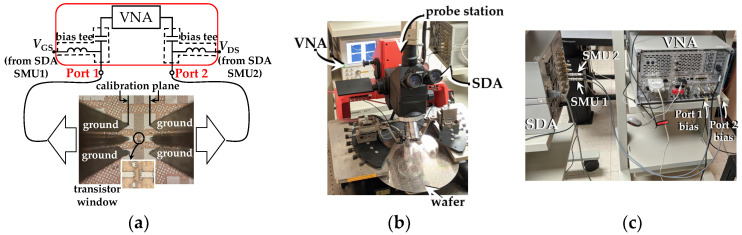
Photographs of the measurement setup: (**a**) DUT while probing, (**b**) probe station, and (**c**) rear panels of the SDA and VNA equipment showing the used configuration.

Several 40 nm n-channel floating body PD SOI MOSFETs included in the same wafer were used in this study. These devices were fabricated by Global Foundries over a high resistivity substrate in a mature RF-SOI 45 nm technology based on [28,29]. For reference, the manufacturer provided, among the specifications, the thickness of the thin oxide *t*_ox_ = 1.18 nm, which is made of silicon oxynitride (SiON). Furthermore, to reduce the negative effect of the gate electrode’s resistance, a double-contact multi-finger layout was employed, and 100 devices were connected in parallel to achieve a total width *W* = 100 μm. As shown in Figure 1a, these devices exhibit a common-source configuration and are embedded within two ground-signal-ground (GSG) pad arrays compatible with RF probes with a pitch of 100 μm. The purpose of this test fixture was to allow the measurement of *S*-parameters at microwave frequencies when considering the gate and drain terminals as the input and output ports, respectively, while the source terminal was the reference. It is important to emphasize that all DC and RF electrical tests outlined in this study were conducted on devices featuring this specific pad configuration.

To perform the DC and RF tests, a vector network analyzer (VNA) and a semiconductor device analyzer (SDA) were interconnected, as described in the schematic shown in Figure 1a. This interconnection allows for applying and collecting RF signals, while biasing the device under test (DUT). In addition, Figure 1b,c show photographs that provide complimentary details about the setup.

### 2.2. Off-State Stress Experiments

**Figure 2:**
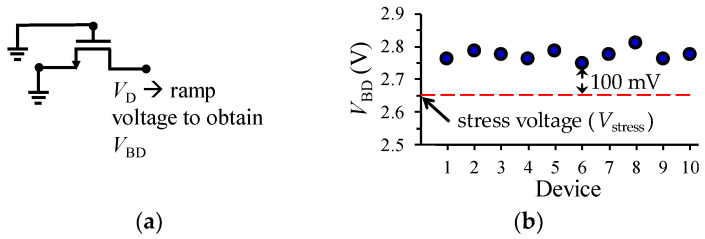
Experiment for defining the drain stress voltage: (**a**) configuration for determining non-conducting breakdown drain voltage for 10 transistors, and (**b**) plot showing the data that allow for establishing *V*_stress_ as a voltage 100 mV below the lowest *V*_BD_ point.

**Figure 3:**
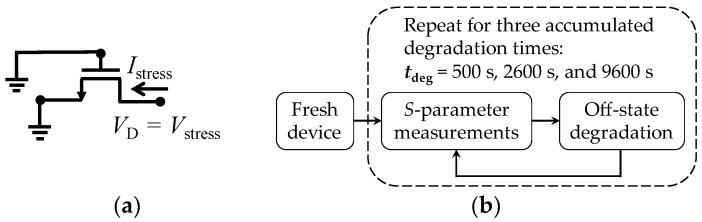
Description of electrical experiments: (**a**) off-state degradation condition, and (**b**) flowchart illustrating the measurement procedure.

To start with the electrical experiments, the nc breakdown drain voltage (*V*_BD_) was obtained for several devices by using the configuration shown in Figure 2a. In this case, a 0 V to 4 V ramp drain voltage (*V*_D_) was applied while the source and gate terminals were grounded; for our purposes, *V*_BD_ is considered as the minimum breakdown voltage obtained for a group of 10 transistors. Afterwards, the DC stress voltage (*V*_stress_) for performing the TDDB test should be defined. In this regard, previous approaches establish the magnitude of *V*_stress_ as high as 0.9 × *V*_BD_. Nonetheless, as illustrated in Figure 2b, here *V*_stress_ was selected smaller than this magnitude to induce observable device degradation at *t*_deg_ intervals in the order of minutes [30]. Based on this fact, with the aim of submitting the fabricated MOSFETs to the stress test, a *V*_stress_ = 2.65 V was applied to the drain terminal of the transistors while the gate and the source terminals were fixed at 0 V, as shown in Figure 3a. This condition was maintained during degradation stages at accumulated times: *t*_deg_ = 500 s, 2600 s, and 9600 s. After each one of these periods of time, *S*-parameter measurements were performed at the bias conditions explained in the following section. To illustrate the experiment cycle, the flowchart in Figure 3b describes the sequence of tests.

### 2.3. S-Parameter Measurements

For performing the two-port *S*-parameter measurements, the VNA setup was calibrated in the range of 50 MHz to 30 GHz using an off-wafer line-reflect-match (LRM) algorithm. Measurements for calibration were conducted on an impedance-standard-substrate (ISS) supplied by the probe manufacturer. This process aimed to define the measurement plane at the tip of the probes and to establish the reference impedance at 50 Ω. In addition, a two-step de-embedding procedure was performed, utilizing measurements from on-wafer “open” and “short” dummy structures with the purpose of eliminating the impact of pad parasitics from the measurements [31].

Since it is the objective of this paper to assess the change of the thin oxide characteristics as the device is degraded, the RF modeling should be kept as simple as possible while capturing the impact of interface trap generation after electrical stress. Therefore, the *S*-parameters were collected under the so-called cold-FET condition, which occurs when the device is biased at a drain-to-source voltage *V*_DS_ = 0 V, and well into the strong inversion region (i.e., the gate-to-source voltage *V*_GS_ is higher than the threshold voltage). This condition offers the advantage of allowing the simplification of the small-signal equivalent circuits for the transistor’s input impedance (*Z*_in_) thanks to the negligible influence of gain effects and the small magnitude of the channel resistance (*R*_ch_) [32]. The equivalent circuit modeling for the MOSFET under this condition is explained below.

## 3. Enhanced Equivalent Circuit Modeling

**Figure 4:**
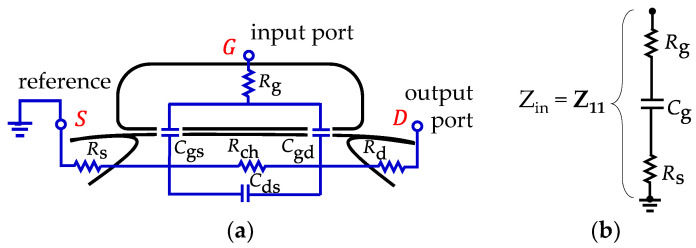
MOSFET conventional small-signal equivalent circuit model under the cold-FET condition: (**a**) two-port model, and (**b**) model for the input port when the drain terminal is left in open circuit condition.

The conventional small-signal model for a MOSFET under the cold-FET condition is shown in Figure 4a, where *C*_gs_, *C*_gd_, and *C*_ds_ are, respectively, the gate-to-source, the gate-to-drain, and the drain-to-source capacitances, and *R*_g_, *R*_d_, and *R*_s_ are the parasitic gate, drain, and source resistances, respectively. Since the devices present a common-source configuration, it is possible to simplify the equivalent circuit for the input impedance to that shown in Figure 4b, where *Z*_in_ = *Z*_11_ is assumed. Thus, the output port, defined at the drain side, is left in an open circuit condition. It should be remarked here that the devices were designed to exhibit an approximately symmetrical structure between the drain and source terminals; hence, at *V*_DS_ = 0 V it is reasonable to assume *C*_gs_ ≈ *C*_gd_ and that the total gate capacitance is *C*_g_ ≈ 2*C*_gs_. Bear in mind, however, that at this point *C*_g_ is considered to be a lossless element, which is an assumption that lacks validity under RF operation, as demonstrated hereafter.

### 3.1. Modeling of Loss Mechanisms on Input Impedance

In accordance with the circuit in Figure 4b, the device’s input impedance can be mathematically expressed as:

(1) $$Z_{\mathrm{in}}=Z_{11}=R_{g}+R_{s}-\;j\frac{1}{\omega C_{g}}$$

where *ω* is the angular frequency, *Z*_11_ belongs to the two-port network *Z*-parameter set, and *j*^2^ = −1. In addition, in Equation (1), the term *R*_ch_/3 associated with the effect of the channel resistance (*R*_ch_) on the input port (see [32]) is neglected due to its small magnitude for short channel devices in strong inversion. On the other hand, observe in Equation (1) that the gate capacitance is assumed to be lossless (e.g., no current is considered to occur within the gate oxide). Regarding this, Equation (1) predicts that the device’s input resistance should exhibit a constant magnitude when plotted versus frequency, given by:

(2) Rin=Re(Zin)=Rg+Rs

Nevertheless, it is well known that *R*_in_ shows a significant dependence on frequency, particularly at relatively low frequencies within the microwave range [33]. In fact, this effect is more accentuated as the gate leakage current (*I_g_*), though the oxide is increased [34]. This is mainly due to the non-negligible loss associated with the gate capacitance.

Actually, there are two main mechanisms that originate energy loss within the gate oxide when operating a MOSFET under alternating current (AC) stimuli. The first one is that associated with the dielectric polarization currents due to the time varying transverse electric field [35]. In this case, the vibration of electric dipoles introduces loss, which rises approximately proportional to frequency. Thus, a conductance *G*_g_ in parallel with *C*_g_ allows for representing this effect, where [36]:

(3) $$G_{g}=\omega C_{g}\tan\delta$$

In this equation, tanδ is the effective loss tangent associated with the dielectric media surrounding the gate electrode, more prominently the gate oxide.

The second loss effect occurring within the oxide is associated with the charging and discharging of existing traps [37]. Hence, since this mechanism exhibits a response limited by the average time for traps to capture and emit carriers (i.e., *τ*), under AC operation, it requires to be represented by a frequency-dependent admittance (*Y*_trap_). An approach to account for this admittance is using a one-pole transfer function defined in terms of the interface trap delay constant (*τ*_it_) and capacitance (*C*_it_) for a single level interface state; this is [38]:

(4) $$Y_{\mathrm{trap}}=\frac{{j\omega C}_{it}}{1+j\omega \tau }$$

In fact, this concept has been applied to propose equivalent circuits for representing border traps in MOS structures [23,25]. In this regard, to better understand the effect of considering this complex function, it is convenient to expand it into real and imaginary parts. This allows for defining the equivalent conductance and capacitance associated with the dynamic trap mechanism, respectively, as:

(5) Gtrap=Re(Ytrap)=ω2τitCit1+(ωτit)2

and

(6) Ctrap=−Im(Ytrap)ω=Cit1+(ωτit)2

Hence, the dynamic charging and discharging of traps within the oxide can be represented by the shunt connection of *G*_trap_ and *C*_trap_, with the gate capacitance *C*_g_ [39,40]. Notice that this implies that the existence of traps within the gate oxide affects not only *R*_in_, but also the transistor input capacitance, defined as:

(7) Cin=−1ωIm(Z11)

Also, bear in mind that traps are randomly distributed within the oxide; thus, Equations (5) and (6) define effective parameters using a first-order relaxation function.

**Figure 5:**
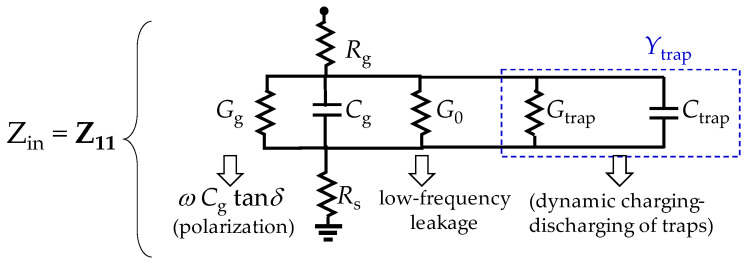
Enhanced model to consider the impact of the gate capacitance loss effects on the input impedance.

To complete the model for the loss mechanisms affecting the gate capacitance, it is necessary to consider that at low frequencies there is still a loss introduced by the gate leakage from the gate electrode to the channel. This is observed even for fresh devices that have not been electrically stressed. However, Equation (5) predicts that there is no conductive path under DC operation (i.e., *G*_trap_ = 0 when *ω* approaches 0). Thus, an additional conductance *G*_0_ is included in the final representation of the input impedance shown in Figure 5. 

### 3.2. Parameter Extraction Methodology

**Figure 6:**
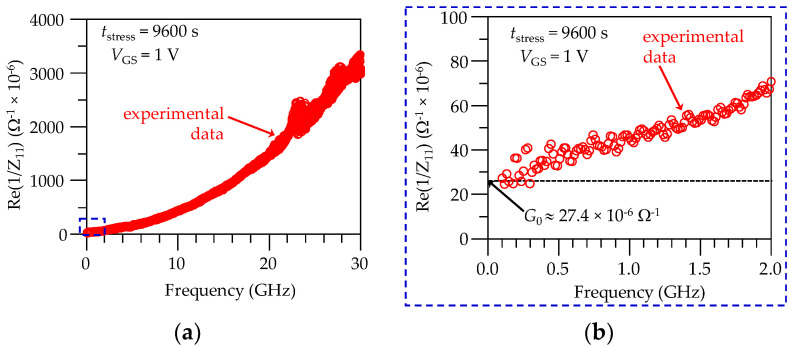
Illustration of the extraction for *G*_0_ from experimental Re(1/*Z*_11_) data: (**a**) curve shown for the full measured range, and (**b**) zoomed-in plot showing the extracted value for *G*_0_.

In order to implement the model in Figure 5, the series resistances *R*_g_ and *R*_s_ are firstly determined from the *S*-parameter measurements at a given bias condition using the method in [22], which also allows for obtaining *C*_g_. Afterwards, it is considered that at frequencies well below 1 GHz, the effect of the polarization currents is negligible. This is because the vibration of the material dipoles is not as high as to represent a noticeable loss and implies that *G*_g_ ≈ 0. Furthermore, at these low frequencies, the expression for the calculation of *G*_0_ can be deduced from the model in Figure 5; this is:

(8) $$G_{0}\approx {Re\left.\left(\frac{1}{Z_{11}-R_{g}-R_{s}}\right)\right|}_{LF}\approx {Re\left.\left(\frac{1}{Z_{11}}\right)\right|}_{LF}$$

where the LF subscript is used to indicate validity at low frequencies; furthermore, the approximation at the extreme left of Equation (8) is used, since 1/*G*_0_≫*R*_g_ + *R*_s_. Figure 6a,b illustrate the extraction of this parameter at *V*_DS_ = 0 V and *V*_GS_ = 1 V for a degradation time *t*_stress_ = 9600 s. For this purpose, the corresponding Z-parameters were obtained from a straightforward two-port S-to-Z network parameter transformation applied to the de-embedded measurements. Notice that the extracted small-signal conductance is *G*_0_ = 27.4×10^−6^ Ω^−1^, which corresponds to a magnitude of the equivalent resistance exhibited by the oxide path at low frequencies of about 36.5 kΩ. This value is within the order of that obtained for degraded multi-finger RF transistors from a DC *I*_G_ versus *V*_G_ curve [24].

**Figure 7:**
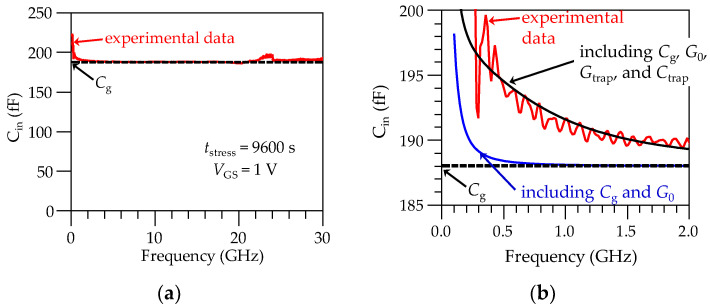
Implementation of the model for *Y*_trap_ from experimental *C*_in_ = –1/*ω*Im(*Z*_11_) data: (**a**) curve for the full measured range, and (**b**) zoomed-in plot showing the model in Figure 5 when considering the different effects.

Now, consider the experimental data for *C*_in_, calculated from Equation (7). As expected, when plotting these data versus frequency in Figure 7a, a significant increase of the input capacitance is observed at relatively low frequencies. This effect is due to the dynamic trapping of carriers, and thus can be represented in the equivalent circuit model by *G*_trap_ and *C*_trap_. In fact, in Figure 7b it is observed that considering only *C*_g_, as typically assumed for characterization and modeling purposes at microwave frequencies [41], predicts a frequency-independent behavior of the input capacitance, since the associated loss mechanisms are neglected. Nonetheless, including the already known *G*_0_ in the model only allows us to achieve a poor agreement with the experimental *C*_in_, as the frequency rises up to the beginning of the microwave range. For this reason, the circuit in Figure 5 is simulated in Keysight Advanced Design System (ADS) to allow implementing the model for *Y*_trap_ by determining only two parameters (i.e., *τ*_it_ and *C*_it_) through a model–experiment correlation; as observed in Figure 7b, the latter model accurately reproduces the experimental data.

**Figure 8:**
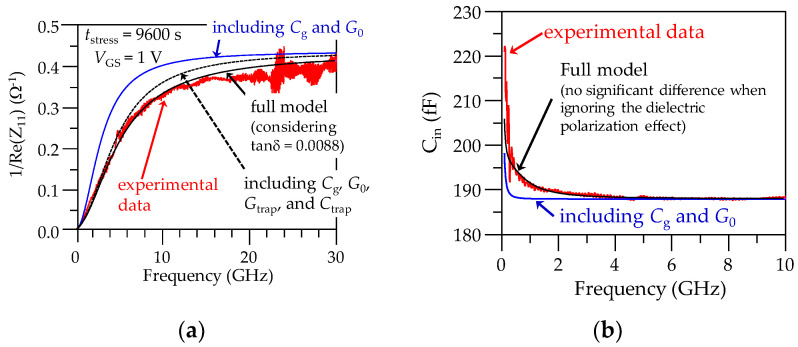
Illustration of the determination of the effective loss tangent associated with the dielectric polarization, which allows for obtaining *G*_g_ = *ω*tan*δC*_g_: (**a**) correlation of the model for the inverse of the input resistance, and (**b**) model–experiment comparison showing that *G*_g_ negligibly impacts the input capacitance.

The only remaining unknown circuit element in the model in Figure 5 is *G*_g_, defined in Equation (3). Since this shunt conductance is approximately proportional to frequency, its effect is mainly observed in the input resistance at high frequencies. Therefore, to allow for observing this effect in the experimental data with detail, it is convenient to correlate the model at high frequencies, considering 1/*R*_in_ = 1/Re(*Z*_11_) data. Figure 8a shows that solely considering *G*_0_ is insufficient to achieve an appropriate correlation, whereas including the *Y*_trap_ components improves the modeling by up to about 5 GHz. Fortunately, tanδ in Equation (3) can be assumed as approximately constant within the microwave range for high-K dielectrics [42]. Thus, Figure 8 shows that using a tanδ = 0.0088 allows us to include *G*_g_ in the equivalent circuit model for achieving excellent agreement with the experimental data, providing a further enhancement of the classical small-signal model for a PD SOI MOSFET under off-state conditions [43]. Moreover, it is important to point out that *G*_g_ plays no significant role in determining the *Y*_trap_ parameters from the *C*_in_ curve in Figure 7b. For this purpose, it is shown in Figure 8b that the simulated curve for *C*_in_ is not sensitive to *G*_g_ within the frequency range at which *G*_trap_ and *C*_trap_ were previously determined.

## 4. Results

**Figure 9:**
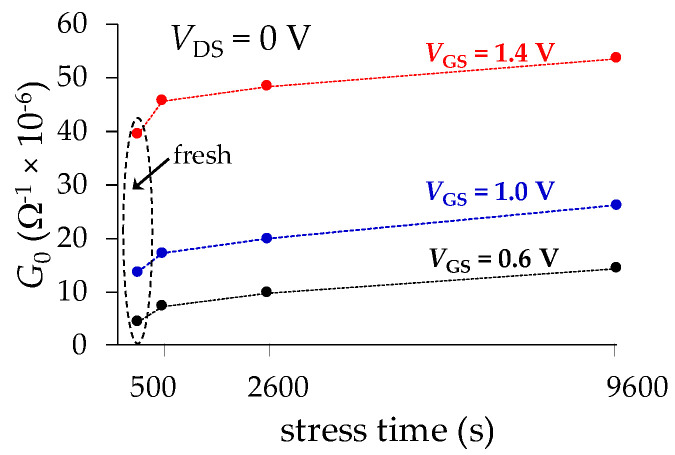
Low-frequency gate oxide conductance extracted from experimental data using the proposal.

For starting the discussion on the experimental results, firstly it should be pointed out that the gate leakage current is obviously increased with *V*_GS_. Indeed, the increase of the vertical electric field promotes the conduction of carriers through the gate oxide, which makes *G*_0_ increase with *V*_GS_ and also with *t*_stress_, as shown in Figure 9. Interestingly, notice that the first points in the versus-*t*_stress_ data shown in this figure indicate that even before the occurrence of nc TDDB, a device exhibits an oxide conductance (i.e., related to the gate leakage) observable at microwave frequencies. 

**Figure 10:**
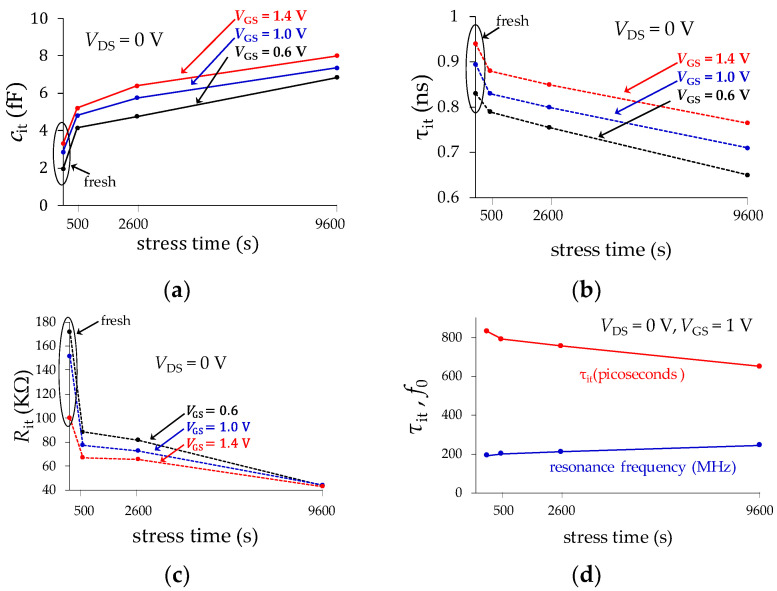
Extracted parameters for the model associated with *Y*_trap_: (**a**) *C*_it_, (**b**) *τ*_it_, (**c**) *R*_it_, and (**d**) the interface resonant frequency calculated from these parameters.

Regarding the model parameters for the admittance of *Y*_trap_, it is expected that the effective capacitance associated with the transistor’s input port is increased as the effect of the dynamic trapping and de-trapping of carriers in the gate oxide is accentuated, which occurs when either *V*_GS_ or *t*_stress_ are increased. This is experimentally verified when plotting *τ*_it_ and *C*_it_ versus *t*_stress_ in Figure 10a,b, respectively, which also evidences that these parameters exhibit more magnitude as *V*_GS_ increases. In other words, as the vertical electric field is more intense, the effect of the loss and delay introduced by the dynamic charging as discharging of traps is more evident, which increases the trap capacitance and conductance. The way that these results can be interpreted is that the applied off-state stress originates performance degradation that is manifested as an increase in the additional capacitance related to the gate oxide. Nevertheless, this increase of capacitance is not contributing to the control of the charge carriers in the channel, but only to the delay in the response of the device. Since there is an intensification of the vertical electric field as *V*_GS_ rises, this phenomenon is accentuated with this voltage. To complement the explanation, the interface trap effective resistance, calculated as *R*_it_ = *τ*_it_/C_it_, is plotted in Figure 10c for the different bias and nc stress conditions considered in our experiments. Since this resistance is an indicator of the AC loss due to dynamic trapping and de-trapping of charge, it is expected to be reduced as the device is more degraded (i.e., the AC conductance of the oxide is increased). Finally, the resonance frequency associated with the trapping-detrapping effect as *f*_it_ = 1/(2π*τ*_it_) is plotted in Figure 10d. Notice that, as the device becomes more degraded, *f*_it_ is shifted down to lower frequencies, making the effect of the dynamic charging of traps evident even far below 1 GHz, which is undesirable under AC operation. 

**Figure 11:**
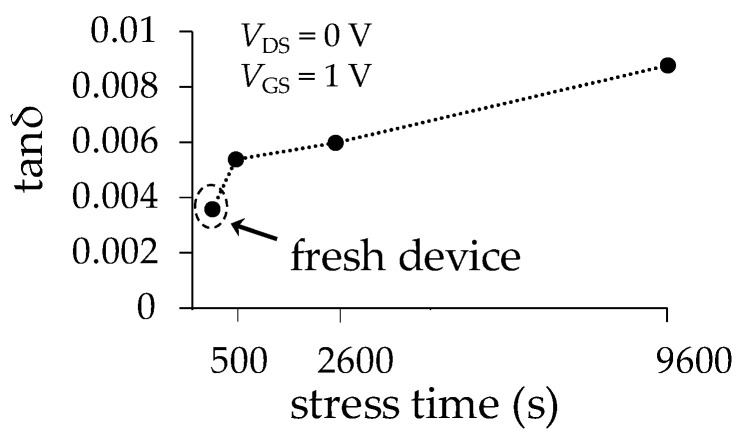
Extracted effective loss tangent data for the dielectric environment.

For the case of the polarization loss, the extracted loss tangent doubles its magnitude after 9600 s of off-state stress when comparing it with that of the device at fresh condition. This result is shown in Figure 11 for the *V*_DS_ = 0 V and *V*_GS_ = 1 V case, and barely noticeable variations are obtained for the other considered gate bias conditions. This suggests that the change in the properties of the thin oxide by the generation of traps is the effect contributing to increasing this type of losses, whereas there is a weak sensitivity to the applied vertical electric field.

**Figure 12:**
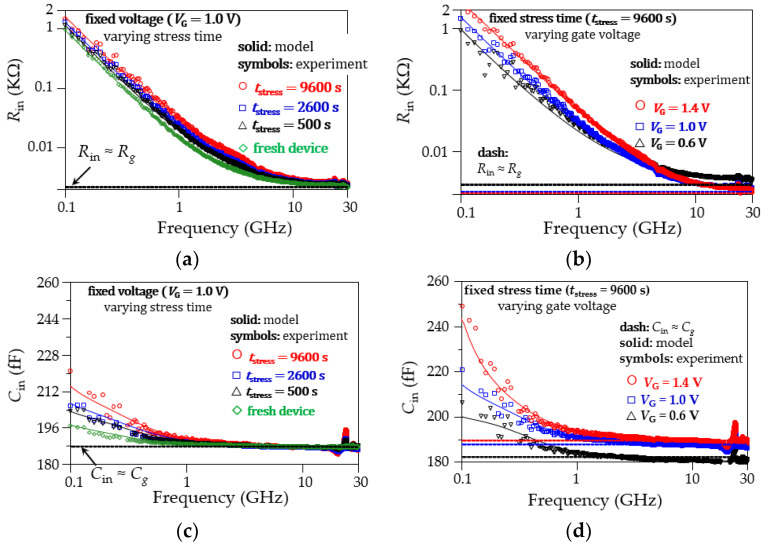
Model–experiment correlations for the input resistance, *R*_in_ = Re(*Z*_11_), and capacitance, *C*_in_ = –1/ωIm(*Z*_11_): (**a**) *R*_in_ at a fixed bias and for different *t*_stress_, (**b**) *R*_in_ at *V*_DS_ = 0 V for the maximum *t*_stress_ and varying *V*_GS_, (**c**) *C*_in_ at a fixed bias and for different *t*_stress_, and (**d**) *C*_in_ at *V*_DS_ = 0 V for the maximum *t*_stress_ and varying *V*_GS_.

As a final form of validation for the proposed modeling approach, curves obtained by simulating the circuit in Figure 5 using the extracted data are confronted with experimental data for the device’s input resistance and capacitance. This is shown in Figure 12a–d. At this point, it is relevant to mention that both *R*_in_ and *C*_in_ exhibit significant frequency dependence due to the loss and delay effects that are occurring within the gate oxide. This dependence is observed even at fresh device conditions, and it is accentuated as the time during which the device is stressed under off-state conditions is increased. We demonstrate that this behavior is accurately predicted when applying the proposed model and parameter extraction methodology. Moreover, the frequency dependence of *R*_in_ and *C*_in_ occurs at relatively low frequencies within the microwave range, when the capacitive reactance of *C*_g_ is not fully dominant. Therefore, it should be highlighted that performing accurate S-parameter measurements below a few gigahertz is important when characterizing these effects using VNA equipment.

A final remark on the application of the proposal is regarding the analysis of the convenience and limitations of the characterized devices when used in specific circuit implementations. In fact, since similar degradation mechanisms are observed in other technologies, effects on the cutoff frequency, gate sub-threshold swing [44], and on devices exhibiting a floating substrate [41] can be explored through simulations using the modeling approach described in this paper.

## 5. Conclusions

Loss and delay mechanisms occurring in the thin gate oxide are distinguished by inspecting the RF input impedance of PD-SOI transistors, since these effects are manifested by increasing the magnitude of the input resistance and capacitance. In fact, the corresponding curves exhibit a significant frequency dependence, specifically below about 3 GHz for devices in a mature 45 nm RF SOI technology, differing for the ideal flat curves expected when assuming lossless gate capacitance conditions. Hence, to contribute to the modeling of the non-ideal behavior of the gate capacitance, here, the dynamic trapping and de-trapping of charges in the oxide, as well as the polarization currents, are identified, quantified, and represented by means of conductances and capacitances. In this regard, the associated model parameters are systematically determined. Moreover, the proposed modeling and characterization methodology shows itself to be consistent when applied to process experimental *S*-parameters performed to devices degraded under non-conducting conditions, and at several gate-to-source voltages. This makes evident the usefulness of RF measurement equipment for assessing the reliability of MOSFETs prone to present significant gate leakage before and after being submitted to electrical stress.

## Data Availability

Data are contained within the article.
